# Effects of eight weeks of an alleged aromatase inhibiting nutritional supplement 6-OXO (androst-4-ene-3,6,17-trione) on serum hormone profiles and clinical safety markers in resistance-trained, eugonadal males

**DOI:** 10.1186/1550-2783-4-13

**Published:** 2007-10-19

**Authors:** Dan Rohle, Colin Wilborn, Lem Taylor, Chris Mulligan, Richard Kreider, Darryn Willoughby

**Affiliations:** 1 Pharmacology Department, Weill Cornell Graduate School of Medical Sciences, 445 East 69th Street, Room 412, New York, NY 10021, USA; 2 Department of Exercise and Sport Science, University of Mary Hardin-Baylor, UMHB Station, 900 College Box 8010, Belton, Texas 76513, USA; 3 Department of Health, Leisure, and Exercise Science, University of West Florida, 11000 University Parkway, # 72-219, Pensacola, FL 32514, USA; 4 Department of Nutrition and Food Science, Colorado State University, Fort Collins, CO 80523, USA; 5 Department of Health, Human Performance, and Recreation, Baylor University, Box 97313, Waco, TX 76798, USA; 6 Institute for Biomedical Studies, Baylor University, Waco, TX 76798, USA

## Abstract

The purpose of this study was to determine the effects of 6-OXO, a purported nutritional aromatase inhibitor, in a dose dependent manner on body composition, serum hormone levels, and clinical safety markers in resistance trained males. Sixteen males were supplemented with either 300 mg or 600 mg of 6-OXO in a double-blind manner for eight weeks. Blood and urine samples were obtained at weeks 0, 1, 3, 8, and 11 (after a 3-week washout period). Blood samples were analyzed for total testosterone (TT), free testosterone (FT), dihydrotestosterone (DHT), estradiol, estriol, estrone, SHBG, leutinizing hormone (LH), follicle stimulating hormone (FSH), growth hormone (GH), cortisol, FT/estradiol (T/E). Blood and urine were also analyzed for clinical chemistry markers. Data were analyzed with two-way MANOVA. For all of the serum hormones, there were no significant differences between groups (p > 0.05). Compared to baseline, free testosterone underwent overall increases of 90% for 300 mg 6-OXO and 84% for 600 mg, respectively (p < 0.05). DHT underwent significant overall increases (p < 0.05) of 192% and 265% with 300 mg and 600 mg, respectively. T/E increased 53% and 67% for 300 mg and 600 mg 6-OXO, respectively. For estrone, 300 mg produced an overall increase of 22%, whereas 600 mg caused a 52% increase (p < 0.05). Body composition did not change with supplementation (p > 0.05) and clinical safety markers were not adversely affected with ingestion of either supplement dose (p > 0.05). While neither of the 6-OXO dosages appears to have any negative effects on clinical chemistry markers, supplementation at a daily dosage of 300 mg and 600 mg for eight weeks did not completely inhibit aromatase activity, yet significantly increased FT, DHT, and T/E.

## Background

Athletes have long been looking for a way to gain an edge in competition, which has lead many to turn to anabolic steroids. Anabolic steroids are defined as testosterone (TST) or derivatives of TST that are used for their ability to create a state of nitrogen retention and increase fat-free mass by stimulating protein synthesis and/or by decreasing protein breakdown. It has been previously thought that anabolic steroids did not cause an increase in muscle size and strength, but now more recent studies have shown the effect that supra-physiological levels of TST and TST derivatives can increase muscle size and strength in males [1-7].

Once produced, TST does not circulate freely in the blood. Rather, total testosterone (TT) is almost 100% bound in blood to proteins with 40% bound to albumin, 40% bound to a β-globulin called sex hormone binding globulin (SHBG), and 17% is bound to other proteins. The small fraction of TST that is not bound is considered the free testosterone (FT) and is the bioactive component of the hormone. Once bound to its androgen receptor, TST can also be converted to dihydrotestosterone (DHT) by the enzyme 5-α reductase. Alternatively, TST can be converted into estradiol through aromatization by the action of the enzyme aromatase.

There are pro-hormone nutritional supplements available, such as androstenedione, that are precursors to TST, and designer androstenedione derivatives such as androstenediol that are purported to support TST production. These compounds are alleged to increase TST, or to increase the concentration of compounds that can act like TST. There are data in young men demonstrating that the acute sublingual ingestion of androstenedione and androstenediol increased FT and TT up to 180 min [8] and 240 min [9] after ingestion. However, these are acute studies with a small window of TST elevation and do not relevantly reflect the manner in which these types of supplements are typically utilized. More appropriately, there are studies demonstrating that the daily oral ingestion of these compounds over the course of several weeks, in conjunction with resistance training [10,11] and otherwise [12] to be ineffective at increasing endogenous TST levels.

However, in the continued attempts to find supplements that elevate testosterone levels, some companies are manufacturing compounds that have no apparent androgenic activity, but are targeted at increasing the endogenous levels of TST by blunting aromatization and subsequent estrogen synthesis. Aromatase inhibiting drugs are not new and have been used for years as a method of preventing and treating various types of cancer. The drugs operate by suppressing estrogen levels and subsequently increasing endogenous free testosterone levels [increased free testosterone/estrogen (T/E) ratio] and the effects of various pharmacologic aromatase inhibitors such as anastrozole and exemestane on the T/E in both young and old men are well documented [13-15].

Nutritional supplements designed with the intent of inhibiting aromatase activity are relatively new to the fitness community. Examples of these supplements are 6-OXO and Novedex XT, and are alleged to act similar to such aromatase inhibiting drugs as formestane. We have recently shown that eight weeks of supplementation with the aromatase inhibiting nutritional supplement Novedex XT (hydroxyandrost-4-ene-6,17-dioxo-3-THP ether and 3,17-diketo-androst-1,4,6-triene) was effective at increasing serum testosterone and DHT, while only displaying slight increases in estrogen levels in young, eugonadal men [16]. Additionally, compounds with the same (androst-4-ene-3,6,17-trione) and very similar (androst-5-ene-4,7,17-trione) structure as 6-OXO have been shown to irreversibly bind to the aromatase enzyme thereby causing a decrease in estradiol production [17,18]. Therefore, use of these aromatase-inhibiting compounds seem to decrease aromatization and subsequent estradiol synthesis, which apparently increases both TST and T/E.

In view of our previous work [16], there is still little data available on the effects of the various nutritional aromatase inhibiting supplements. Therefore, the purpose of the study was threefold and was to determine the efficacy of an eight week oral supplementation period with either 300 mg/day or 600 mg/day of 6-OXO on: 1) serum hormone levels, 2) serum and urinary clinical safety markers and systemic hemodynamic effects, and 3) serum hormone, serum and urinary clinical safety markers, and systemic hemodynamic effects after a 3-week washout period following both supplementation protocols.

## Methodology

### Participants

Sixteen apparently healthy, recreationally-active males with a mean age of 26.6 ± 4.9 years, height of 180.2 ± 6.3 cm, body fat of 14.9 ± 4.8 %, and body weight of 87.3 ± 13.2 kg served as participants in the study. All participants were cleared for participation by passing a mandatory medical screening. Participants with contraindications to exercise as outlined by the American College of Sports Medicine and/or who had consumed any nutritional supplements (excluding multi-vitamins) such creatine monohydrate or various androstenedione derivatives or pharmacologic agents such as anabolic steroids two months prior to the study were not allowed to participate. All eligible subjects signed a university-approved informed consent document. Additionally, all experimental procedures involved in this study conformed to the ethical considerations of the Helsinki Code.

### Testing sessions

Testing sessions were performed at week 0 and after weeks 1, 3, 8, and 11 in which blood and urine samples were obtained and where body composition, serum hormones, blood and urinary clinical safety markers, and systemic hemodynamic safety markers were evaluated.

### Body composition assessment

Total body mass (kg) was determined on a standard dual beam balance scale (Detecto Bridgeview, IL). Percent body fat, fat mass, and fat-free mass were determined using DEXA (Hologic 4200 W, Waltham, MA). Quality control calibration procedures were performed on a spine phantom (Hologic X-CALIBER Model DPA/QDR-1 anthropometric spine phantom) and a density step calibration phantom prior to each testing session. Total body water and compartment-specific fluid volumes were determined by bioelectric impedance analysis (Xitron Technologies Inc., San Diego, CA).

### Blood and urine collection

Venous blood samples were obtained from an antecubital vein into a 10 ml collection tube. Blood samples were allowed to stand at room temperature for 10 min and then centrifuged. The serum was removed and frozen at -80°C for later analysis. Urine samples were obtained in mid-stream into a collection container using a standard collection protocol. Urine samples were frozen at -80°C for later analysis. Blood and urine samples were obtained after a 12-hour fast and standardized to the same time of day for each sample.

### Supplementation protocol

Based on the premise that there were no muscle performance measurements to be made in the study, such as muscle strength and power, that could otherwise generate a so-called "placebo effect," the decision was made not to utilize a placebo group. Participants were equally divided, matched by age and body mass, and then randomly assigned in double-blind fashion to an eight-week supplementation protocol consisting of the daily oral ingestion of either 300 mg or 600 mg of 6-OXO [androst-4-ene-3,6,17-trione (ErgoPharm, Champaign, IL)]. For the 300 mg group, (n = 8; total body mass = 79.3 ± 13.2 kg, fat-free mass = 67.1 ± 7.9 kg; body fat = 14.7 ± 5.4 %) 100 mg was ingested in the morning with breakfast and 200 mg was ingested with the evening meal. For the 600 mg group, (n = 8; total body mass = 81.1 ± 13.3 kg, fat-free mass = 69.0 ± 12.1 kg; body fat = 15.0 ± 4.2 %) 300 mg was ingested in the morning with breakfast and 300 mg was ingested with the evening meal. For days where no exercise occurred, the supplements were ingested in the same timely fashion. After the supplementation period, a three-week washout period was required during which neither supplement was ingested. Upon analysis of serum testosterone from the baseline blood samples at week 0, it was confirmed that all participants completing the study were eugonadal [10–30 nmol/L (27–107 ng/ml)][19].

### Physical activity, dietary intake records, and supplementation compliance

During both the supplementation and washout periods the participants' physical activity and dietary intake were not supervised; however, it was required that all participants keep detailed physical activity and dietary records and not change their routine dietary habits or level of physical activity throughout the course of the study. As such, participants were required to keep weekly physical activity records and four-day dietary records during weeks 0, 1, 3, 8, and 11 and turn in their physical activity and dietary records during each testing session. Each participant returned all of their dietary and physical activity evaluations at the required time points for a 100% compliance rate. The four-day dietary recalls were evaluated with the Food Processor dietary assessment software program (ESHA Research, Salem, OR) to determine the average daily macronutrient consumption of fat, carbohydrate, and protein. In an effort to ensure compliance to the supplementation protocol, participants were supplied with the appropriate number of capsules to be ingested during the time between testing sessions 1, 3, and 8. Upon reporting to the lab at each of the respective testing sessions, participants returned the empty containers and a capsule count was performed if necessary.

### Reported side effects from supplements

At the last four testing sessions, participants reported by questionnaire whether they tolerated the supplement, supplementation protocol, as well as report any medical problems/symptoms they may have encountered throughout the study.

### Hemodynamic clinical safety markers

At each testing session, participants assumed a supine position for 15 minutes and had their heart rate (HR), systolic blood pressure (SBP), and diastolic blood pressure (DBP) determined to assess the hemodynamic safety of supplementation with 6-OXO. Heart rate was determined by use of a Polar heart rate monitor (Polar, San Ramon, CA), and blood pressure was assessed with a mercurial sphygmomanometer using standard procedures.

### Blood and urinary clinical markers

The serum clinical chemistry variables glucose, total protein, blood urea nitrogen, creatinine, BUN/creatinine ratio, uric acid, AST, ALT, CK, LDH, GGT, albumin, globulin, sodium, chloride, calcium, carbon dioxide, total bilirubin, alkaline phosphatase, triglycerides, cholesterol, HDL, and LDL were determined with a Dade Dimension RXL clinical chemistry analyzer (Dade-Behring, Inc., Newark, DE). The whole blood hematological variables, hemoglobin, hematocrit, red blood cell counts, MCV, MCH, MCHC, RDW, neutrophils, lymphocytes, monocytes, eosinophils, and basophils, were determined with an Abbott Cell Dyne 3500 hematology analyzer (Abbott Laboratories, Chicago, IL). The urinary variables glucose, ketones, blood, protein, nitrite, bilirubin, leukocyctes, specific gravity, pH, urobilinogen were analyzed with a Bayer Clinitek 200 Plus urine analyzer (Bayer Diagnostics, Tarrytown, NY).

### Serum hormones

Serum TT, FT, DHT, estradiol, estrone, estriol, SHBG, LH, growth hormone (GH), cortisol (Diagnostics Systems Laboratories, Webster, TX), and FSH (Alpco Diagnostics, Windham, NH) using enzyme-linked immunoabsorbent assays (ELISA) and enzyme-immunoabsorbent assays (EIA) with a Wallac Victor-1420 microplate reader (Perkin-Elmer Life Sciences, Boston, MA), and the assays were performed at a wavelength of either 450 or 405 nm, respectively. The average specificity for all assays was 3.5 pg/ml, and in all cases the intra-assay and inter-assay variances were < 10%. Additionally, the amount of cross-reactivity between androstenedione and FT, TT, and DHT was 0.06%, 0.09%, and 1.9%, respectively.

### Statistical analysis

Due to the likelihood of the body composition, serum hormones, and serum and urinary clinical chemistry marker dependent variables being related to one another, statistical analyses were performed by utilizing three separate repeated-measures two-factor [treatment groups (2) × time point (5)] mixed methods multivariate analysis of variance (MANOVA). The use of MANOVA also reduces the risk of Type I errors, by controlling for alpha level that could result with the use of repeated analyses of variance (ANOVA). Box M tests were performed to test for differences in covariance matrices. Bartlett's Test of Sphericity were performed to test that the variance and covariance matrix of the dependent variables was circular in form which would allow for accurate interpretation of univariate ANOVAs. Levene's Test of Equality of Error was performed to test for equality of variance for each dependent variable. Further analysis of the main effects for Group and Test were performed by separate one-way ANOVAs and by using Sidak pair-wise comparisons. Significant between-group differences were then determined by employing the Tukey HSD Post Hoc Test when the variances were equal and a Games-Howell Post Hoc Test was used when the variances were not equal. The changes from post- to pre-training for each criterion variable were then analyzed with a one-way ANOVA. Non-significant trends (p < .10) were reported as partial Eta squared to illustrate the effect size, where an effect size of 0.51 is relatively strong, 0.24 is moderate and is 0.17 relatively weak [20]. All statistical procedures were performed using SPSS 13.0 software and a probability level of < 0.05 was adopted throughout.

## Results

### Physical activity and dietary intake records

All 16 participants appeared to have exhibited 100% compliance with the supplement protocol, and were able to complete the required dosing regimen and testing procedures with no side effects from both the 300 mg and 600 mg doses of 6-OXO. In addition, subjective analysis of the physical activity evaluations revealed that none of the participants had any noticeable changes in their level of physical activity over 11-week period. There were no significant differences between groups (p > 0.05) in total daily caloric or macronutrient intake of carbohydrates, protein, and fats over the course of the 11 weeks (data not shown).

### Body composition

Table 1 shows the measured mean ± SD values for the body composition variables. The results revealed no significant group × time interaction (p = 0.884) or main effect for test (p = 0.581) or time (p = 0.748) indicating that there were no significant differences in body composition values.

**Table 1:** Body Composition Variables

	300 mg					600 mg				
Week	0	1	3	8	11	0	1	3	8	11

ECF (L)	19.6 ± 2.5	19.9 ± 3.1	19.6 ± 2.4	19.5 ± 2.3	20.0 ± 2.5	19.8 ± 3.1	20.2 ± 3.3	20.0 ± 2.7	20.2 ± 3.3	21.2 ± 3.1
ICF (L)	30.0 ± 5.1	30.3 ± 5.3	30.0 ± 5.0	29.2 ± 4.2	29.9 ± 4.8	29.5 ± 6.2	29.7 ± 5.0	29.9 ± 4.5	30.6 ± 6.4	32.5 ± 5.8
TBW (L)	49.5 ± 7.4	50.2 ± 8.1	49.6 ± 7.2	48.7 ± 6.2	49.9 ± 7.0	49.3 ± 9.2	49.8 ± 8.2	49.9 ± 7.2	50.8 ± 9.6	53.6 ± 8.7
Fat Mass (kg)	12.2 ± 6.3	12.4 ± 6.2	12.5 ± 6.2	13.0 ± 5.5	13.0 ± 5.8	12.1 ± 3.5	12.5 ± 3.3	12.4 ± 3.5	12.1 ± 3.2	12.6 ± 3.4
Fat-Free Mass (kg)	64.5 ± 7.7	64.6 ± 8.0	64.1 ± 7.5	63.9 ± 8.7	64.0 ± 8.1	69.0 ± 12.1	68.9 ± 12.4	68.8 ± 11.1	69.9 ± 12.0	70.1 ± 13.2
Total Body Mass (kg)	79.3 ± 13.2	79.6 ± 13.7	79.3 ± 12.9	79.5 ± 13.0	78.8 ± 13.8	81.1 ± 13.3	81.5 ± 13.8	81.3 ± 12.8	82.0 ± 13.5	82.7 ± 14.7
Body Fat (%)	14.7 ± 5.4	14.9 ± 5.2	15.1 ± 5.3	15.9 ± 4.7	15.8 ± 4.9	15.0 ± 4.2	15.4 ± 4.0	15.3 ± 3.9	14.8 ± 3.6	15.3 ± 3.8

No significant differences in body composition were observed during the 11 weeks (p > 0.05).

### Whole blood clinical safety markers

Table 2 shows the measured mean ± SD values for the whole blood clinical chemistry markers. The results demonstrated no significant group × time interaction (p = 0.829) or main effects for test (p = 0.567), indicating that there were no significant differences in whole blood clinical safety markers over the course of the study. However, the results did show a significant main effect for group, indicating that the 600 mg group had higher baselines values for hematocrit (p = 0.030), absolute monocytes (p = 0.027), and absolute basophils (p = 0.018) that persisted throughout the study, and was apparently independent of 6-OXO supplementation.

**Table 2:** Hemodynamic Safety Markers

	300 mg					600 mg				
Week	0	1	3	8	11	0	1	3	8	11

Heart Rate (bpm)	64 ± 7.6	58 ± 4.7	61 ± 5.1	61 ± 5.6	64 ± 8.7	57 ± 6.2	58 ± 6.7	59 ± 7.2	61 ± 3.0	64 ± 8.6
SBP (mmHg)	116 ± 13.0*	114 ± 9.8*	117 ± 12.3*	117 ± 10.7*	116 ± 8.8*	75 ± 8.7	74 ± 9.3	73 ± 8.1	70 ± 7.9	77 ± 5.6
BBP (mmHg)	109 ± 6.4	107 ± 5.2	113 ± 6.9	115 ± 11.4	114 ± 4.8	69 ± 8.2	74 ± 4.5	71 ± 9.4	76 ± 9.9	74 ± 6.3

* Denotes significant main effects for groups (p < 0.05). Results indicated that the 300 mg group had significant higher baseline values that persisted throughout the study, and was apparently independent of 6-OXO supplementation.

### Serum clinical safety markers

Table 3 shows the measured mean ± SD values for the serum clinical chemistry markers. Results showed no significant group × time interaction (p = 0.815) or main effects for test (p = 0.671) indicating that there were no significant differences in whole blood clinical safety markers over the course of the study. However, the results did show significant main effects for group and revealed that the 600 mg group was shown to have significantly higher baseline values for total cholesterol (p = 0.02), low density lipoprotein (p = 0.01), blood urea nitrogen (p = 0.016), GGT, calcium, total blood protein, and albumin (p = 0.01) that persisted throughout the study, and was apparently independent of 6-OXO supplementation.

**Table 3:** Whole Blood Clinical Chemistry Markers

	300 mg					600 mg				
Week	0	1	3	8	11	0	1	3	8	11

WBC (K/μL)	5.3 ± 1.6	5.0 ± 1.1	5.3 ± 1.7	5.2 ± 2.0	4.5 ± 1.5	5.5 ± .96	4.6 ± .91	5.0 ± 1.0	4.9 ± .67	5.1 ± .84
RBC (M/μL)	5.0 ± .51	5.0 ± .50	4.6 ± .84	5.0 ± .37	4.9 ± .46	5.2 ± .37	5.1 ± .33	5.2 ± .24	4.9 ± 1.2	5.3 ± .30
Hemoglobin (g/dL)	15 ± 1.1	15 ± 1.1	15 ± 1.2	15 ± .59	15 ± 1.0	15 ± .95	15 ± .93	15 ± .63	15 ± .66	15 ± .78
Hematocrit (%)	44 ± 3.5	44 ± 3.7	44 ± 3.6	45 ± 2.4	43 ± 3.3	46 ± 3.0*	44 ± 2.3*	45 ± 1.9*	46 ± 2.1*	46 ± 2.7*
MCV (fL)	88 ± 2.8	88 ± 2.8	88 ± 2.4	89 ± 2.4	89 ± 2.9	87 ± 2.7	86 ± 2.8	87 ± 2.6	87 ± 2.3	87 ± 2.5
MCH (pg)	30 ± 1.2	30 ± 1.3	30 ± 1.0	30 ± 1.3	30 ± 1.0	29 ± 1.0	29 ± .95	29 ± .98	29 ± 1.1	29 ± .78
MCHC (g/dL)	34 ± .69	34 ± .76	34 ± .64	33 ± .78	34 ± .81	33 ± .60	34 ± .85	33 ± .56	33 ± .61	34 ± .60
Neutrophils	2.9 ± 1.0	2.5 ± .43	2.9 ± 1.1	2.8 ± 1.0	2.3 ± 1.1	2.8 ± .52	2.4 ± .47	2.6 ± .66	2.5 ± .29	2.9 ± 1.1
Lymphocytes	1.7 ± .51	1.9 ± .84	1.7 ± .62	1.8 ± .87	1.6 ± .69	2.0 ± .39	1.6 ± .39	1.8 ± .50	1.7 ± .44	1.8 ± .44
Monocytes	.45 ± .12	.39 ± .12	.44 ± .12	.36 ± .07	.37 ± .14	.43 ± .14*	.38 ± .04*	.39 ± .13*	.40 ± .09*	.44 ± .12*
Eosinophils	.12 ± .06	.13 ± .08	.11 ± .05	.12 ± .06	.12 ± .06	.14 ± .08	.14 ± .09	.16 ± .10	.16 ± .09	.16 ± .07
Basophils	.06 ± .02	.05 ± .01	.05 ± .01	.05 ± .01	.05 ± .02	.07 ± .02*	.06 ± .02*	.07 ± .02*	.07 ± .01*	.06 ± .01*

* Denotes significant main effects for groups (p < 0.05) indicating that the 600 mg group had higher baselines values that persisted throughout the study, and was apparently independent of 6-OXO supplementation.

### Urine clinical safety markers

Table 4 shows the measured mean ± SD values for the urine clinical chemistry markers. The results revealed no significant group × time interaction (p = 0.794) or main effect for test (p = 0.543) or time (p = 0.693) indicating that there were no significant differences in urine clinical safety markers over the course of the study.

**Table 4:** Serum Clinical Chemistry Markers

	300 mg					600 mg				
Week	0	1	3	8	11	0	1	3	8	11

Triglyceride (mg/dl)	86.8 ± 29.5	98.5 ± 46.1	105.3 ± 29.5	110.3 ± 58.8	85.9 ± 41.9	117.5 ± 60.2	115.5 ± 50.5	116.6 ± 40.5	110.9 ± 43.4	97.8 ± 36.1
Cholesterol (mg/dl)	177.0 ± 38.2	182.1 ± 26.1	173.9 ± 32.2	175.0 ± 22.6	166.1 ± 20.7	194.6 ± 24.9*	185.1 ± 22.3*	201.1 ± 20.8*	201.6 ± 37.8*	199.5 ± 31.0*
HDL (mg/dL)	50.1 ± 12.5	51.3 ± 10.9	53.6 ± 17.6	49.4 ± 7.9	48.3 ± 8.9	53.0 ± 4.7	53.8 ± 9.8	58.5 ± 11.9	53.9 ± 18.7	57.0 ± 18.3
LDL (mg/dL)	107.9 ± 26.9	112.0 ± 18.8	105.4 ± 23.5	104.4 ± 22.8	100.6 ± 16.8	115.9 ± 21.5*	111.4 ± 21.7*	124.8 ± 22.0*	127.1 ± 33.5*	121.4 ± 25.2*
GGT (U/L)	23.9 ± 5.7	23.8 ± 4.6	23.1 ± 4.8	27.3 ± 7.9	26.0 ± 7.0	32.3 ± 9.6*	33.6 ± 11.2*	33.6 ± 7.8*	38.8 ± 11.8*	36.4 ± 8.3*
LDH (U/L)	127.0 ± 19.4	130.6 ± 23.3	125.4 ± 19.6	114.5 ± 25.8	130.0 ± 33.0	125.5 ± 17.4	128.0 ± 18.0	132.8 ± 23.4	132.1 ± 24.6	140.6 ± 32.6
Uric Acid (g/dl)	6.1 ± 1.7	5.8 ± 1.1	5.7 ± 0.9	5.2 ± 1.0	5.5 ± 1.4	6.1 ± 1.3	6.1 ± 1.6	6.5 ± 1.2	5.7 ± 1.0	5.7 ± 1.3
Glucose mg/dl	94.5 ± 9.4	95.8 ± 10.3	97.6 ± 10.4	94.0 ± 8.2	93.9 ± 9.6	95.1 ± 8.4	94.3 ± 6.5	100.5 ± 6.1	105.1 ± 17.1	89.9 ± 14.8
BUN (mg/dL)	16.9 ± 2.9	19.4 ± 5.0	20.6 ± 4.5	18.5 ± 3.0	19.4 ± 3.6	21.1 ± 7.6*	22.3 ± 5.1*	22.5 ± 3.8*	19.8 ± 3.5*	21.3 ± 2.4*
Creatinine (mg/dl)	1.4 ± 0.1	1.4 ± 0.2	1.4 ± 0.2	1.3 ± 0.2	1.3 ± 0.3	1.4 ± 0.2	1.4 ± 0.2	1.5 ± 0.2	1.5 ± 0.2	1.3 ± 0.3
Calcium (mg/dl)	12.5 ± 1.7	14.2 ± 3.2	14.8 ± 3.0	14.9 ± 2.1	15.2 ± 3.4	15.6 ± 4.4*	16.4 ± 3.4*	14.9 ± 2.8*	13.7 ± 2.6*	17.0 ± 3.0*
Total Protein g/dL	9.8 ± 0.5	9.9 ± 0.4	9.9 ± 0.6	9.8 ± 0.5	9.5 ± 0.4	10.1 ± 0.6*	10.1 ± 0.7*	10.8 ± 0.7*	10.4 ± 0.7*	10.2 ± 0.9*
Albumin g/dL	7.4 ± 0.6	7.7 ± 0.4	7.8 ± 0.6	7.4 ± 0.5	7.3 ± 0.5	7.9 ± 0.9*	8.0 ± 0.9*	8.5 ± 1.0*	8.3 ± 0.9*	8.1 ± 0.9*
Total Bilirubin (mg/dl)	4.7 ± 0.5	4.8 ± 0.4	4.9 ± 0.5	4.6 ± 0.5	4.6 ± 0.3	5.0 ± 0.5	5.1 ± 0.5	5.4 ± 0.6	5.3 ± 0.6	5.1 ± 0.6
ALP (U/L)	0.8 ± 0.3	0.8 ± 0.6	1.0 ± 0.4	0.9 ± 0.5	0.9 ± 0.6	0.6 ± 0.3	0.6 ± 0.2	0.9 ± 0.4	0.8 ± 0.3	0.8 ± 0.2
AST (U/L)	73.9 ± 13.3	76.3 ± 12.1	73.1 ± 12.8	70.5 ± 15.7	64.0 ± 19.9	77.1 ± 21.3	76.1 ± 21.0	79.5 ± 19.4	76.4 ± 19.0	63.1 ± 27.8
ALT (U/L)	24.4 ± 9.6	24.5 ± 9.3	21.3 ± 6.8	18.3 ± 6.6	30.3 ± 25.1	22.6 ± 6.5	27.3 ± 5.5	30.0 ± 11.7	24.5 ± 8.0	26.3 ± 10.0
CK (U/L)	28.6 ± 11.5	25.6 ± 5.5	22.4 ± 7.2	28.4 ± 10.8	27.1 ± 5.5	28.1 ± 14.2	25.0 ± 7.2	23.8 ± 6.7	28.9 ± 15.6	32.6 ± 10.7

* Denotes significant main effects for groups (p < 0.05) indicating that the 600 mg group had higher baselines that persisted throughout the study, and was apparently independent of 6-OXO supplementation.

### Hemodynamic clinical safety markers

Table 5 shows the measured mean ± SD values for the hemodynamic clinical safety markers. There was no significant group × time interaction (p = 0.725) or main effect for test (p = 0.583) demonstrating no significant differences in hemodynamic measures over the course of the study. However, the results did show a significant main effect for group and revealed that the 300 mg group had a significantly higher baseline SBP (p = 0.041) that persisted throughout the study, and was apparently independent of 6-OXO supplementation.

**Table 5:** Urine Clinical Safety Markers

	300 mg					600 mg				
Week	0	1	3	8	11	0	1	3	8	11

Glucose	0 ± 0	0 ± 0	0 ± 0	0 ± 0	0 ± 0	0 ± 0	0 ± 0	0 ± 0	0 ± 0	0 ± 0
Ketones	0 ± 0	0 ± 0	0 ± 0	0 ± 0	0 ± 0	0 ± 0	0 ± 0	0 ± 0	0 ± 0	0 ± 0
Blood	0 ± 0	0 ± 0	0 ± 0	0 ± 0	0 ± 0	0 ± 0	0 ± 0	0 ± 0	0 ± 0	0 ± 0
Protein	0 ± 0	0 ± 0	0 ± 0	0 ± 0	0 ± 0	0 ± 0	0 ± 0	0 ± 0	0 ± 0	0 ± 0
Nitrite	0 ± 0	0 ± 0	0 ± 0	0 ± 0	0 ± 0	0 ± 0	0 ± 0	0 ± 0	0 ± 0	0 ± 0
Bilirubin	0 ± 0	0 ± 0	0 ± 0	0 ± 0	0 ± 0	0 ± 0	0 ± 0	0 ± 0	0 ± 0	0 ± 0
Leukocytes	0 ± 0	0 ± 0	0 ± 0	0 ± 0	0 ± 0	0 ± 0	0 ± 0	0 ± 0	0 ± 0	0 ± 0
Specific Gravity	1.02 ± 0.01	1.02 ± 0.01	1.01 ± 0.01	1.02 ± 0.01	1.02 ± 0.01	1.02 ± 0.01	1.02 ± 0.01	1.02 ± 0.01	1.02 ± 0.01	1.02 ± 0.01
pH	5.5 ± 0.76	5.6 ± 0.58	5.9 ± 0.90	5.4 ± 0.68	5.3 ± 0.53	5.8 ± 1.13	5.8 ± 1.13	5.8 ± 1.13	5.8 ± 1.13	5.8 ± 1.13
Urobilinogen (E.U./dl)	0.2 ± 0.0	0.2 ± 0.0	0.2 ± 0.0	0.2 ± 0.0	0.2 ± 0.0	0.2 ± 0.0	0.2 ± 0.0	0.2 ± 0.0	0.2 ± 0.0	0.2 ± 0.0

No significant differences were observed (p > 0.05). A zero reading indicates that there were no traceable amounts of markers present. Readings that were above a zero reading indicated a trace amount and are otherwise indicated by numbers 1, 2, and 3.

### Serum hormones

Table 6 shows the mean values ± SD for all the hormones over the course of the study. There was no significant group × test interaction (p = 0.882) for any of the hormones measured (p = 0.882). However, there were significant main effects for group for SHBG (p = 0.022), LH (p = 0.01), and FSH (p = 0.014) with a trend towards significance in cortisol (p = 0.053, effect size = 0.052). Pair-wise comparisons indicated that the 600 mg group had lower levels of SHBG and LH, and higher levels of FSH. These differences existed at baseline and persisted throughout the duration of the study and were, therefore, independent of 6-OXO supplementation.

**Table 6:** Serum Hormone Levels

	300 mg					600 mg				
Week	0	1	3	8	11	0	1	3	8	11

TT (ng/ml)	4.7 ± 2.1	6.2 ± 1.4	5.6 ± 2.0	5.8 ± 2.1	5.0 ± 1.0	3.9 ± 1.2	5.6 ± 1.5	5.8 ± 1.3	5.4 ± 1.9	6.29 ± 5.0
‡ FT (pg/ml)	17.2 ± 8.8	37.1 ± 33.03*	30.13 ± 17.3*	30.5 ± 16.9*	18.2 ± 10.6	22.7 ± 13.0	39.2 ± 13.2*	45.1 ± 27.0*	40.6 ± 36.1*	22.4 ± 9.8
‡ DHT (ng/mL)	0.9 ± 0.4	3.1 ± 1.8*	2.8 ± 1.4*	2.4 ± 1.3*	1.0 ± 0.3	0.9 ± 0.2	3.0 ± 1.3*	4.4 ± 3.8*	2.8 ± 1.7*	1.7 ± 1.2
Estradiol (ng/mL)	89.6 ± 86.2	112.3 ± 124.2	121.2 ± 125.6	107.1 ± 106.6	101.8 ± 103.8	69.1 ± 23.6	71.4 ± 35.9	85.5 ± 49.4	75.8 ± 47.9	78.3 ± 54.5
‡ Estriol (pg/ml)	0.04 ± 0.03	0.04 ± 0.04	0.05 ± 0.07	0.05 ± 0.06	0.06 ± 0.05	0.03 ± 0.04	0.04 ± 0.05	0.04 ± 0.03	0.05 ± 0.03	0.04 ± 0.02
‡ T/E	0.2 ± 0.1	0.3 ± 0.3*	0.3 ± 0.1*	0.3 ± 0.2*	0.2 ± 0.1	0.3 ± 0.2	0.6 ± 0.4*	0.5 ± 0.1*	0.5 ± 0.5*	0.3 ± 0.2
Estrone (pg/ml)	334.1 ± 89.5	428.8 ± 122.*	400.9 ± 135.4*	397.2 ± 166.0*	323.5 ± 89.3	268.8 ± 43.7	425.3 ± 137.0*	431.7 ± 141.6*	365.6 ± 92.0*	308.8 ± 101.6
† SHBG (nmol/L)	128.9 ± 36.6	135.7 ± 33.5	127.4 ± 37.3	137.0 ± 42.5	132.5 ± 43.5	130.6 ± 26.6*	119.5 ± 32.6*	106.9 ± 17.6*	101.4 ± 29.5*	112.9 ± 35.8*
†LH (mIU/ml)	6.6 ± 4.2	8.9 ± 5.6	6.0 ± 3.2	6.3 ± 3.0	5.4 ± 2.4	3.2 ± 1.8*	4.5 ± 2.1*	4.3 ± 1.9*	4.1 ± 2.6*	3.9 ± 2.6*
† FSH (mIU/ml)	0.03 ± 0.04	0.04 ± 0.06	0.03 ± 0.03	0.06 ± 0.06	0.03 ± 0.05	0.1 ± 0.2*	0.09 ± 0.3*	0.03 ± 0.07*	0.08 ± 0.2*	0.2 ± 0.4*
Cortisol (μg/dl)	23.0 ± 7.1	24.3 ± 7.3	23.6 ± 4.1	19.5 ± 8.9	22.4 ± 5.0	24.5 ± 6.5	25.4 ± 4.1	25.5 ± 3.6	26.3 ± 8.1	24.9 ± 4.9
GH (pg/ml)	46.9 ± 9.7	59.7 ± 150.7	62.4 ± 44.1	82.2 ± 64.2	107.6 ± 284.4	56.8 ± 114.1	49.7 ± 72.1	119.4 ± 306.2	63.4 ± 43.7	165.8 ± 254.1

† Significant main effect for Group (p < 0.05). ‡ Significant main effect for Test (p < 0.05). * Significantly different from baseline (Week 0) (p < 0.05).

There was a significant main effect for Test for FT (p = 0.017), DHT (p = 0.006), T/E (p = 0.025), and estrone (p = 0.007), along with a trend for significance for TT (p = 0.062, effect size = 0.068). Compared to baseline, post-hoc tests showed FT levels to be higher at week 1 (p = 0.016) and week 3 (p = 0.019), and week 8 (p = 0.037). DHT levels were significantly higher at week 1 (p = 0.026), week 3 (p = 0.004), and week 8 (p = 0.014). T/E levels were significantly higher at week 1 (p = 0.034), week 3 (p = 0.018), and week 8 (p = 0.37). Estrone was significantly higher at week 1 (p = 0.003) and week 3 (p = 0.007), with a trend towards also being higher at week 8 (p = 0.057, effect size = 0.056).

## Discussion

In this study, we sought to determine the effects of 6-OXO supplementation provided at a daily dosage of 300 mg and 600 mg for eight weeks on body composition, serum hormones, and clinical safety markers. There were no adverse side effects reported from the participants and no significant changes in hemodynamic measures and in clinical chemistry markers measured in whole blood, serum, or urine during the course of the study suggesting that 6-OXO at the dosages investigated for a period of eight weeks appears safe within the confines of the markers assessed.

In regard to body composition, neither dose of 6-OXO demonstrated any significant improvement in fat mass or fat-free mass over the course of the study. Even with significant increases in FT and DHT, this furthers indicates that 6-OXO supplemented at these dosages for eight weeks did not decrease fat mass or cause an anabolic response by increasing muscle mass. Increased serum androgens levels have been shown to stimulate lipolysis due to increases in the activity of hormone sensitive lipase [21]; however, relative to the dosage and/or the duration of ingestion of 6-OXO on serum androgens in the present study, neither had any effect on body composition. In our previous study, eight weeks of supplementation with 72 mg/day of the nutritional aromatase inhibitor Novedex XT had no effect on fat-free mass, but was effective at producing a modest, but significant 3.5% decrease in fat mass when compared to placebo [16].

For the serum hormones, the only significant changes that occurred over the course of the study that were 6-OXO dependent were for FT, DHT, estrone, and T/E. FT and DHT underwent overall increases of 90% and 192% for 300 mg 6-OXO and 84% and 265% for 600 mg, respectively, while T/E increased 53% and 67% for 300 mg and 600 mg 6-OXO, respectively. However, for FT, DHT, and T/E there were no significant differences between groups, suggesting 300 mg and 600 mg of 6-OXO to be equally as effective in increasing androgen levels. In addition, by the end of the three-week washout period, the levels of these hormones (and all others) had returned to normal levels. The FT, DHT, and T/E data is in agreement with previous research, which showed an increase in TT with ingestion of an aromatase inhibitor [19,22,23], with 6-OXO (unpublished data) [24], and with our previous study with Novedex XT where we showed average increases of 283%, 625%, and 566% for TT, FT, and DHT [16]. Relative to the ability of 6-OXO to inhibit aromatization by way of our serum hormone markers, estrone underwent overall increases of 22% and 52% for 300 mg and 600 mg, respectively, and estradiol underwent overall increases of 27% and 12% for 300 mg and 600 mg, respectively. These data indicate that aromatase activity was not completely inhibited by 6-OXO throughout the eight-week period.

It seems logical that DHT concentration would increase concomitantly with elevations in TST because DHT is a TST metabolite. Relative to the change in FT and the significant increases in DHT over the eight-week period, the present data suggest a role for 6-OXO in up-regulating the activity of the 5α-reductase enzyme. Unlike TST, DHT is a non-aromatizable androgen [13]. Because 6-OXO is a type I steroidal aromatase inhibitor, it is assumed that this supplement would completely inhibit aromatase activity, thereby leading to elevations in endogenous TST. However, our results suggest the contrary; 6-OXO does elevate TST and DHT levels without the complete inhibition of serum aromatase activity. As a result, it is conceivable that the apparent TST aromatization occurring was in part responsible for the observed elevations in estradiol and estrone. Aromatase catalyzes the conversion of TST to estradiol, of androstenedione to estrone, and of 16α-hydroxylated dehydroepiandrosterone to estriol [25]. The purported mechanism for an increase in TST with aromatase inhibition has been reported as a decrease in estradiol levels that leads to feedback to the hypothalamus to stimulate TST-induced increases in estradiol [26,27]. This would infer that in order for an increase in TST to occur, a decrease in estradiol would have to be seen, and this is not what happened in this study.

Regarding SHBG, the levels did not change over the course of the study. This suggests that the effects of 6-OXO on FT to be somewhat independent of the circulating levels of SHBG since at least 95% of circulating testosterone is bound to SHBG at any one time. Interestingly, however, the 600 mg group had lower SHBG concentrations at baseline and throughout the course of the study suggesting this to be independent of the specific dose of 6-OXO.

The results of this study indicate that eight weeks of 6-OXO supplementation had no effect on body composition or clinical safety markers, but incompletely inhibited aromatase activity and significantly increased endogenous DHT levels that were attenuated after a three-week washout period. Therefore, while neither of the 6-OXO dosages appears to have any negative effects on clinical chemistry markers, supplementation at a daily dosage of 300 mg and 600 mg for eight weeks did not completely inhibit aromatase activity, yet significantly increased FT, DHT, and T/E.

## Competing interests

The author(s) declare that they have no competing interests.

## Authors' contributions

DR participated in the design of the study, coordination and data acquisition, and assisted in performing the statistical analysis and drafting the manuscript. CW, LT, CM participated in the data acquisition. RK participated in the design of the study and assisted in performing the statistical analysis. DSW conceived the study, developed the study design, secured the funding for the project, assisted and provided oversight for all data acquisition and statistical analysis, assisted in drafting the manuscript, and served as the faculty mentor for the project.
